# Technology Based Cognitive Behavioral Therapy On Psychological Distress: Exploring Health, Pain, And Activity

**DOI:** 10.1093/geroni/igab046.3139

**Published:** 2021-12-17

**Authors:** Avani Shah, Martin Morthland, Forrest Scogin

**Affiliations:** 1 University of Alabama, Tuscaloosa, Alabama, United States; 2 University of Alabama, University of Alabama, Alabama, United States

## Abstract

This randomized controlled trial investigates two technologically based self-administered cognitive behavioral depression treatments (CBT) on psychological distress in older adults. Health may change the ability to participate in types of activities, thereby impacting mental well-being and treatment response. The aims of this research are 1) to understand the impact of technologically based cognitive behavioral treatment on psychological distress 2) explore how health, pain, and activity engagement may affect treatment response. Fifty one participants recruited were randomized to one of 3 groups: audio-based cognitive behavioral therapy, computer-based cognitive-behavioral therapy, and control group. The combined treatment groups are compared to the control group. Health was examined in multiple ways; the Vulnerable Elders Scale-13 score ; (Saliba et al., 2001); and a reported chronic pain condition. For overall psychological distress, improvement on the Brief Symptom Inventory General Severity Index (GSI; Derogatis & Spencer, 1983) scores from baseline to post-treatment indicated treatment response. The California Older Person’s Pleasant Events Scale (COPPES; Rider, Gallagher-Thompson, & Thompson, 2004) measured activity engagement. While controlling for the Time 1 GSI score, an ANOVA revealed a significant difference in psychological distress between the CBT treatment group and control group F(1, 43) =4.22, p=.046. A linear regression analysis with the VES-13 score and GSI baseline score as predictors and the GSI posttreatment score as the dependent variable, found that health did not significantly predict psychological distress outcomes. Observation of the descriptives and these analyses suggest that CBT can impact psychological distress, potentially even with variations in health and pain.

